# Neuroanatomical features in soldiers with post-traumatic stress disorder

**DOI:** 10.1186/s12868-016-0247-x

**Published:** 2016-03-31

**Authors:** D. Sussman, E. W. Pang, R. Jetly, B. T. Dunkley, M. J. Taylor

**Affiliations:** Department of Diagnostic Imaging, The Hospital for Sick Children, 555 University Avenue, Toronto, ON M5G 1X8 Canada; Division of Neurology, Neuroscience and Mental Health Program, The Hospital for Sick Children, 555 University Avenue, Toronto, ON M5G 1X8 Canada; Directorate of Mental Health, Canadian Forces Health Services, Ottawa, ON Canada

## Abstract

**Background:**

Posttraumatic stress disorder (PTSD), an anxiety disorder that can develop after exposure to psychological trauma, impacts up to 20 % of soldiers returning from combat-related deployment. Advanced neuroimaging holds diagnostic and prognostic potential for furthering our understanding of its etiology. Previous imaging studies on combat-related PTSD have focused on selected structures, such as the hippocampi and cortex, but none conducted a comprehensive examination of both the cerebrum and cerebellum. The present study provides a complete analysis of cortical, subcortical, and cerebellar anatomy in a single cohort. Forty-seven magnetic resonance images (MRIs) were collected from 24 soldiers with PTSD and 23 Control soldiers. Each image was segmented into 78 cortical brain regions and 81,924 vertices using the corticometric iterative vertex based estimation of thickness algorithm, allowing for both a region-based and a vertex-based cortical analysis, respectively. Subcortical volumetric analyses of the hippocampi, cerebellum, thalamus, globus pallidus, caudate, putamen, and many sub-regions were conducted following their segmentation using Multiple Automatically Generated Templates Brain algorithm.

**Results:**

Participants with PTSD were found to have reduced cortical thickness, primarily in the frontal and temporal lobes, with no preference for laterality. The region-based analyses further revealed localized thinning as well as thickening in several sub-regions. These results were accompanied by decreased volumes of the caudate and right hippocampus, as computed relative to total cerebral volume. Enlargement in several cerebellar lobules (relative to total cerebellar volume) was also observed in the PTSD group.

**Conclusions:**

These data highlight the distributed structural differences between soldiers with and without PTSD, and emphasize the diagnostic potential of high-resolution MRI.

## Background

Posttraumatic stress disorder (PTSD) is an anxiety disorder that can develop after exposure to psychological trauma, such as military combat. It is characterized by a cluster of emotional and behavioural symptoms, including the inability to regulate memory, as well as difficulty with emotional processing, attention, and language. Its prevalence among soldiers returning from deployment in Afghanistan and the Middle East is estimated to be 10–20 % [52]. Unfortunately, standard routine clinical brain imaging often shows no discernible evidence of any brain damage [40], and thus, cannot accurately validate the condition. Advanced neuroimaging holds diagnostic and prognostic potential that could help identify reliable structural biomarkers of PTSD that could help in treatment [45] and in understanding the aetiology of the disorder.

Previous structural neuroimaging studies have focused primarily on the prefrontal cortex [30], due to its role in inhibitory control, and the hippocampi [23, 32], due to their involvement in episodic memory and stress. However, results have been heterogeneous, with some showing a bilateral volumetric reduction to the hippocampi [23, 24] and others failing to replicate such reduction [3, 19, 26, 33, 39, 56]. This inconsistency in the literature may be due in part to the inclusion of a wide age range and the types of stressors that brought about PTSD in patients, as well as the use of control groups that are not always matched on exposure to trauma, e.g., civilians compared to front-line soldiers.

Other brain structures have received comparatively less attention, but the available studies report a reduction in volume in the anterior cingulate cortex [29, 57], caudate [25], and insula [8, 25], as well as cortical thinning of the superior and middle frontal gyri, and the inferior and superior frontal gyri [22]. Studies have indicated that the cerebellum might also play a role in anxiety disorders [15]. Yet, to date, no study has been published on cerebellar morphology in combat-related PTSD. The recent finding of reduced cerebellar volume in paediatric patients with PTSD [16] highlights the need to determine the possible role of cerebellar changes in the adult condition. To the best of our knowledge, no single study investigated subcortical, cortical, and cerebellar neuroanatomy in a cohort of soldiers with PTSD. The current study provides thorough analyses of all of these brain structures, with high resolution Magnetic Resonance Imaging (MRI), comparing soldiers with PTSD with carefully-matched combat-exposed control soldiers without PTSD.

## Methods

This study was approved by the Research Ethics Boards of The Hospital for Sick Children and Defence Research and Development Canada, and was conducted in accordance with their guidelines. Written informed consent was obtained from all participants.

### Participants

Forty-seven male active-duty soldiers from the Canadian Armed Forces who served in Afghanistan were recruited to this study: 23 soldiers with a PTSD diagnosis (“PTSD” group: mean age ± SD = 37.3 ± 6.8 years; range 26–48 years), while the remaining 24 soldier controls did not have PTSD (“Control” group: mean age ± SD = 32.9 ± 4.5 years; range 27–40 years).

Soldiers with PTSD were assessed using a semi-structured psychometric interview and diagnosed by a Canadian Forces psychiatrist at a Canadian Forces Operational Trauma and Stress Support Centre and had the primary diagnosis of PTSD. Potential participants with PTSD were sent a letter inviting them to participate in the study, and their decision did not affect the care they received. In all participants with PTSD, onset of the disorder was traced to an operationally-related traumatic event (Category A1). Co-morbid diagnosis in these participants included depression (69.5 %) and other anxiety disorders (18.2 %). Control soldiers were recruited through advertisements posted at Canadian Forces bases in southern Ontario. They were matched with participants with PTSD based on their years of military service, experience, and education levels, and were not included in the study if they had any co-morbid condition. Furthermore, they were screened to exclude a diagnosis of PTSD or childhood trauma. Whilst no explicit reports of Category A1 traumatic-events were recorded in the control group, they served in similar front-line combat roles, and were therefore assumed to have witnessed similarly stressful events as those who did develop PTSD.

Exclusion criteria for both groups included standard neuroimaging safety exclusions, uncorrected vision, having any history of premorbid neurological, psychological or psychiatric disorders, a history of traumatic brain injury (mild, moderate, or severe, and including concussion which is considered a subtype of mild TBI), if they were taking anti-convulsant medications, benzodiazepines and GABA antagonists, or had significant artefacts in their MRI scan. The Wechsler Abbreviated Scale of Intelligence (WASI) was also administered to all Control and PTSD group participants.

### MRI acquisition

Structural brain images were collected from all soldiers using a 3T Siemens Trio MRI scanner (MAGNETOM Tim Trio, Siemens AG, Erlangen, Germany) with a 12-channel head coil. A T1-weighted 3D sagittal magnetization-prepared rapid gradient echo (MP-RAGE) sequence was utilized, which provided 1 mm isotropic voxels, a FOV of 192 × 240 × 256 mm with a 256 × 256 matrix and 192 slices. Sequence parameters included: TR = 2300 ms, TE = 2.96 ms, TI = 900 ms, and FA = 9°. During image acquisition head stabilization and motion restriction was attained using foam padding.

### Image processing

#### Cortical analysis

The CIVET pipeline [62] was used for linearly registering all MRIs into a common 3-dimensional space, correcting for RF inhomogeneity artefacts [12, 50], and classifying cortical regions based on their physiological category: grey matter (GM), white matter (WM), and cerebrospinal fluid [62]. This classification was conducted in a two-step process; with (1) discrete tag points, and (2) partial volume information for the different tissue classes [31]. Grey and white matter surfaces were then produced with the Constrained Laplacian Anatomical Segmentation using Proximities (CLASP) method [31], and used in calculating the cortical surface area (SA). A more accurate identification of grey and white matter surface boundary [31] was created by expanding the white matter surfaces until they reached the grey matter or cerebrospinal fluid surface boundary. The result was 4 surfaces (2 per hemisphere) each containing 40,962 vertices. These surfaces were registered to the Montreal Neurological Institute International Consortium for Brain Mapping 152 (MNI ICBM152) surface template, allowing results to be compared between groups. Surface boundary distance was also utilized in computing the cortical thickness (CT), and together with SA, it was used in calculating the cortical volume (CV) [28, 34]. CT was analysed both with a lobe-based approach, using the 78 brain regions segmented using the AAL atlas [53], and with a vertex-based approach, using all 81,924 vertices.

### Analysis of the hippocampi, cerebellum, and basal-ganglia

The cerebellum, hippocampi, and basal ganglia were segmented in all MR images using the Multiple Automatically Generated Templates (MAGeT) algorithm [7], which utilized manually segmented images as atlases. Five such atlases were used for the cerebellum and hippocampi, and one atlas was used for the basal ganglia. An arbitrary subset of the MR images, which are designated as “templates”, are pair-wise registered to each of the atlases to create multiple anatomical segmentations (masks), yielding a template library consisting of 105 labeled atlases per structure. These atlases are then averaged, and the most frequently occurring segmentation label per voxel is retained, resulting in a more accurate final anatomical segmentation. This procedure is known as “voxel voting” [13]. The automatically segmented cerebellar, hippocampi, and basal ganglia structures for each brain were thereafter used for calculating and comparing the average structural volumes between groups. Dividing the structural volumes by the total brain volume, as computed through the sum of the grey matter, white matter and cerebrospinal fluid volumes in CIVET, yields a measure of the relative volume occupied by each structure.

The segmentation identified both the anatomical structures as well as sub-fields within those structures. Thirteen cerebellar sub-fields were segmented: lobule 1–2, lobule 3, lobule 4, lobule 5, lobule 6, Crus 1, Crus 2, lobule 7b, lobule 8a, lobule 8b, lobule 9, lobule 10, white matter. Five hippocampal sub-fields were segmented: CA1, subiculum, CA4/dentate gyrus, CA2/3, stratum radiatum/lacunosum/moleculare. Finally, the thalamus, globus pallidus, caudate, and putamen were segmented. Sub-fields in the right and left hemisphere were summed, and the combined volume is reported and compared between groups in the analyses.

### Statistical analysis

Analysis of Variance (ANOVA) was used to test the dependence of total brain volume, GM volume, WM volume, CT, SA, CV, cerebellar and sub-cortical volumes on Group and Age. Multiple comparisons within each ANOVA were accounted for through the False Discovery Rate (FDR) technique [2]. This technique was also used to correct the results of the vertex-based cortical thickness analysis. FDR values <15 % counted as significant, and in those cases the specific values are quoted along with the results. Data values are reported in the form of mean ± standard deviation.

## Results

### IQ and cerebral parameters

 Shown in Table 1 are group averages for estimates of IQ (WASI) and cerebral parameters for both the PTSD and control groups, with inferential test-statistics for between-groups comparisons. IQ was found to be significantly lower in PTSD than controls (t = −2.05, p < 0.05), whilst grey matter (GM) volume was significantly increased (t = 2.15, p < 0.05). Furthermore, mean cortical thickness was decreased in the PTSD compared with Control participants (t = −2.63, p < 0.05). No significant differences were found in total brain volume, white matter volume, total surface area, or total cortical volume (p > 0.05).

**Table 1:** IQ scores (WASI) and cerebral parameters for the PTSD and control groups, showing mean and ±1 standard deviation, as well as inferential tests and p-values

N	PTSD	Control	t-Test statistic	p-value
	23	24		
WASI score	109.1 ± 13.5*	117.4 ± 14.2	−2.05	*0.047*
Total brain volume (10 × 5 mm^3^)	31.37 ± 2.13	31.07 ± 2.64	0.42	0.674
Grey matter volume (10 × 5 mm^3^)	14.24 ± 1.45*	13.35 ± 1.35	2.15	*0.037*
White matter volume (10 × 5 mm^3^)	9.95 ± 0.93	10.23 ± 1.12	−0.93	0.357
Total surface area (10 × 5 mm^2^)	1.93 ± 0.11	1.89 ± 0.16	0.83	0.412
Mean cortical thickness (mm)	3.20 ± 0.12*	3.28 ± 0.12	−2.63	*0.011*
Total cortical volume (10 × 5 mm^3^)	5.06 ± 0.43	5.14 ± 0.58	−0.51	0.61

Italics indicates significant differences between groups

* Significant differences between groups

### Lobe-based analysis

A lobe-based analysis of cortical thickness showed diffuse reduced thickness for the PTSD group compared with Controls in a wide variety of regions. The affected regions (those with significant differences) are listed in Table 2, along with their respective thicknesses and effect sizes (at either the FDR level <10 or <5 %). There was neither an age effect, nor surface area difference found between the groups (p > 0.05). The affected regions were mostly found in the frontal and temporal lobes, with no preference for laterality.

**Table 2:** Cortical thickness in (mm) of regions showing significant group differences (with mean ±1 standard deviation)

Conical region	Thickness in PTSD group (mm)	Thickness in control group (mm)	t-Test statistic	FDR (≤%)
Left precentral gyrus	2.84 ± 0.16	2.98 ± 0.16	3.05	5
Right precentral gyrus	2.71 ± 0.18	2.90 ± 0.18	3.67	5
Left superior frontal gyrus, dorsolateral	2.97 ± 0.16	3.09 ± 0.15	2.84	10
Right superior frontal gyrus, dorsolateral	2.92 ± 0.15	3.06 ± 0.15	3.29	5
Left middle frontal gyrus	3.05 ± 0.19	3.16 ± 0.15	2.29	10
Right middle frontal gyrus	3.02 ± 0.13	3.14 ± 0.13	3.08	5
Left supplementary motor area	3.27 ± 0.18	3.40 ± 0.17	2.57	10
Right supplementary motor area	3.25 ± 0.17	3.38 ± 0.17	2.79	10
Left superior frontal gyrus, medial	3.25 ± 0.16	3.37 ± 0.18	2.45	10
Right superior frontal gyms, medial	3.10 ± 0.15	3.22 ± 0.19	2.53	10
Left superior frontal gyrus, medial orbital	3.15 ± 0.15	3.25 ± 0.16	2.26	10
Right calcarine fissure and surrounding cortex	2.89 ± 0.15	2.99 ± 0.15	2.23	10
Left lingual gyrus	3.00 ± 0.11	3.11 ± 0.15	2.61	10
Left inferior occipital gyrus	3.06 ± 0.14	3.17 ± 0.19	2.32	10
Left postcentral gyrus	2.57 ± 0.13	2.70 ± 0.15	3.37	5
Right postcentral gyrus	2.53 ± 0.14	2.67 ± 0.14	3.43	5
Left supramarginal gyrus	3.20 ± 0.15	3.34 ± 0.19	2.71	10
Right angular gyrus	3.06 ± 020	3.19 ± 0.17	2.36	10
Right resell gyrus	3.26 ± 0.17	3.40 ± 0.19	2.67	10
Left superior temporal gyrus	3.21 ± 0.16	3.34 ± 0.15	3.07	5
Right superior temporal gyrus	3.23 ± 0.18	3.37 ± 0.17	2.67	10
Left temporal pole: superior temporal gyrus	3.83 ± 0.24	4.01 ± 0.21	2.74	10
Left middle temporal gyrus	3.24 ± 0.18	3.36 ± 0.15	2.65	10
Left temporal pole: middle temporal gyrus	3.66 ± 0.30	3.85 ± 0.24	2.28	10
Left inferior temporal gyrus	3.31 ± 0.17	3.42 ± 0.13	2.42	10
Right inferior temporal gyrus	3.32 ± 0.20	3.45 ± 0.15	2.45	10

There was no significant effect of age on any structure

### AAL-guided analysis

**Fig. 1:**
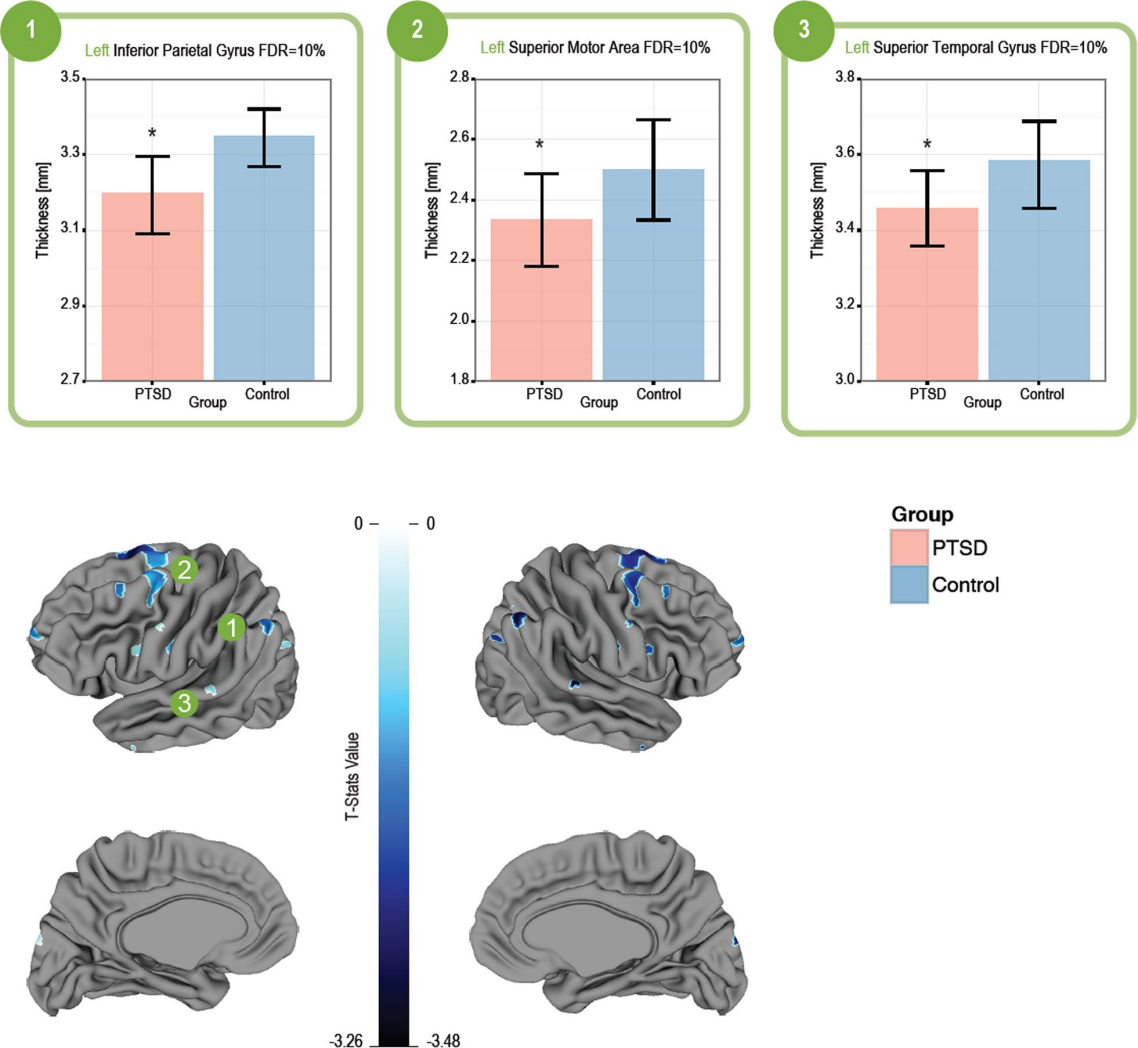
Group differences in AAL-guided cortical thickness analysis. *Bar plots* of the mean and ±1 standard error for cortical thickness in the regions from T-statistic maps highlighting regions with significant group effects of cortical thickness (PTSD n = 23, Control n = 24), as analyzed with a vertex-based analysis. Cool colours on the anatomical images indicate a relative thinning in the cortex for PTSD compared with Controls. The *bar graphs* of selected voxels are plotted, and show significant thinning in the left inferior parietal gyrus (**1**), the left superior motor area (**2**), and the left superior temporal gyrus (**3**); *Asterisk* denotes significant differences using unpaired t-tests with FDR = 10 %

Information about local differences within cerebral regions defined by the AAL atlas was computed using the vertex-based cortical-thickness analysis. This analysis revealed significant group differences, indicating the presence of localized thinning in several regions. T-statistics maps of these results are accompanied by bar-graphs shown in Fig. 1. Graphed regions (shown in the bar charts) include the left inferior parietal gyrus (1), the left superior motor areas (2), and the left superior temporal gyrus (3), which were thinner in the PTSD compared to the Control group (FDR < 10 %).

### Sub-cortical and cerebellar volumes

**Fig. 2:**
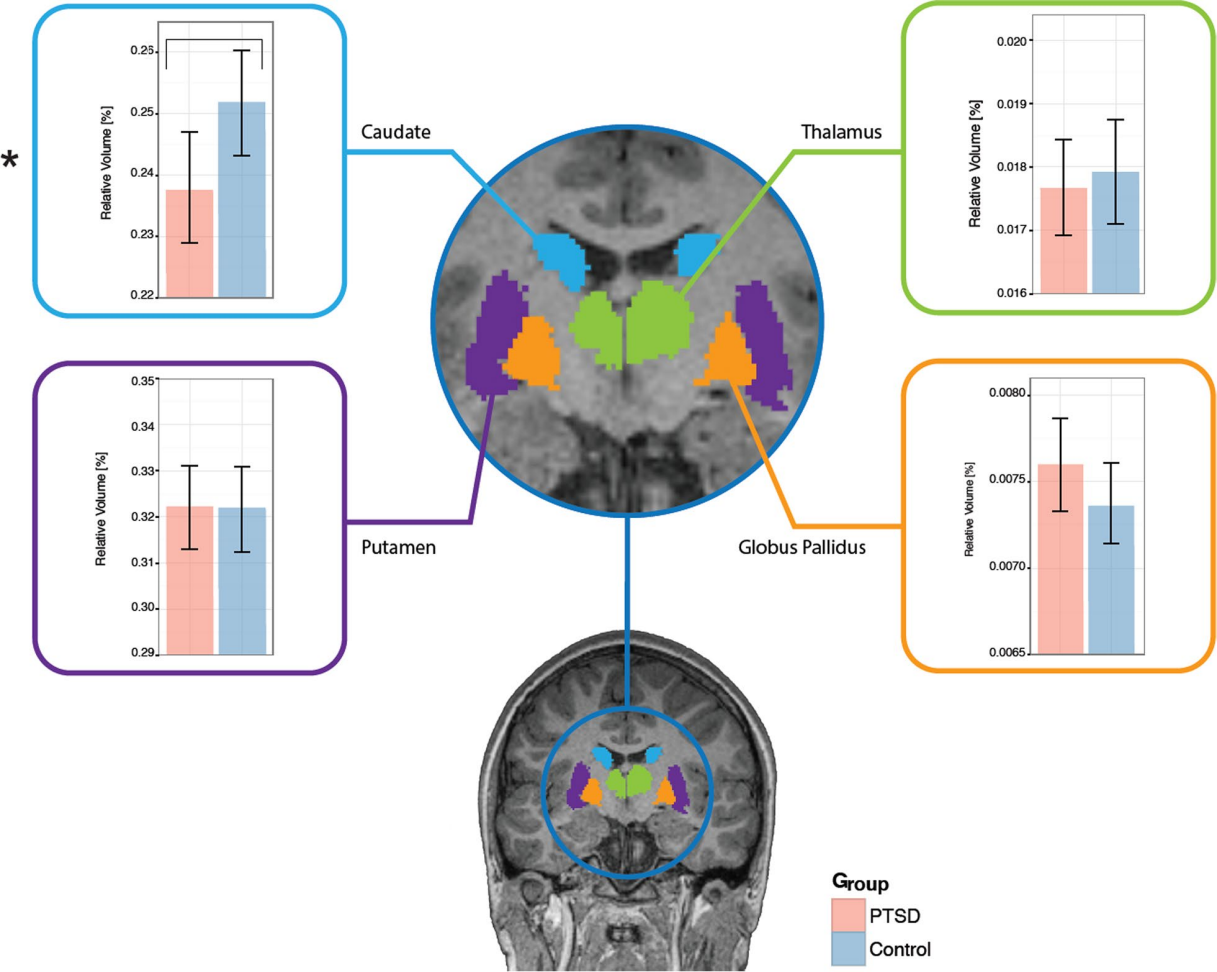
Group differences in the relative volume of subcortical structures for PTSD and control soldiers (*bar graphs* show mean and ±1 standard error). Structural colour maps: *blue* caudate, *green* thalamus, *purple* putamen, *orange* globus pallidus. Significant differences (denoted by *asterisk*) were found in the caudate, with decreased relative volume in the PTSD compared with Control group. No other basal ganglia structure was found to be significantly different between the groups. Vertical axis of graphs shows relative volume in %

A volumetric analysis of sub-cortical and cerebellar structures revealed a number of significant differential effects when comparing our groups, with some regions showing decreased volume in PTSD, whilst others were larger in our clinical group. Specifically, the caudate was found to be significantly smaller in relative volume in the PTSD group (0.238 ± 0.023 %) compared with the Control group (0.252 ± 0.029 %), shown in Fig. 2. No hippocampal region was found to be significantly different between the groups, or the thalamus, putamen, or globus pallidus (all p’s > 0.05).

**Fig. 3:**
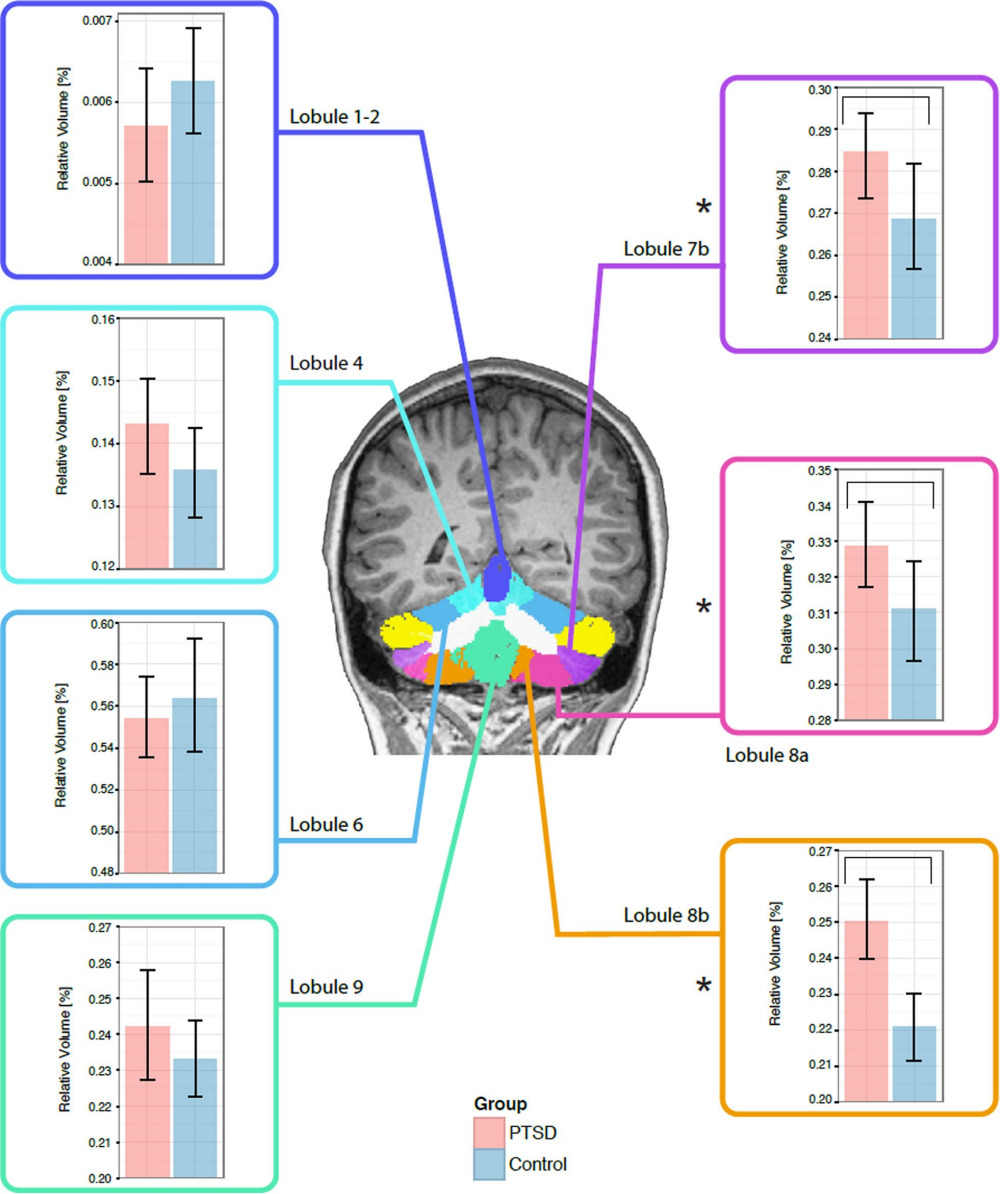
Group differences in the relative volume of cerebellar structures for PTSD and control soldiers (*bar graphs* show mean and ±1 standard error). Division colour maps: *dark blue* Lobule 1–2, *light blue* lobule 4, *blue* lobule 6, *green* lobule 9, *purple* lobule 7b, *pink* lobule 8a, *orange* lobule 8b. Significant differences between the groups (denoted by *asterisk*) were observed in the relative volume of the cerebellum in a number of regions, with enlargement found in lobules 7b, 8a and 8b in the PTSD compared with the Control group. Vertical axis of the graphs show relative volume in %

For the cerebellar structures, a significant group effect was observed for the relative volume of cerebellar lobules 7b, 8a and 8b, which were all significantly larger in relative volume in the PTSD compared with the Control group (FDR = 5–10 %), which is shown in Fig. 3. A group-by-age interaction effect was found for the relative volume of cerebellar lobules 3 and 5, Crus 1 and 2, the right cerebellum, as well as the total cerebellum volume. In all cases, the PTSD group had a smaller volumes compared to those of the Controls.

## Discussion

### Summary

PTSD is a serious neuropsychiatric disorder with no established structural biomarkers for diagnosis. Thus far, neuroimaging studies have focused on either cortical or subcortical structures, but did not conduct both of these analyses on a single cohort. The present study provides a thorough neuro-morphological evaluation of total brain volume, cortical thickness, cortical surface area, as well as the relative volumes of the hippocampi, caudate, globus pallidus, putamen, thalamus, and cerebellum. Results from soldiers with PTSD were compared to those from age- and experience-matched soldier controls. We found that the PTSD diagnosis is accompanied by a slight decrease in the IQ test, reduced cortical thickness, primarily in the frontal and temporal gyri, decreased relative volumes of the caudate, but relative enlargement in several lobules within the cerebellum.

### Behavioural

The WASI test showed a slightly yet significant lower average score for the PTSD group. While the WASI test is generally used for estimation of IQ, performance on it is susceptible to emotional state. Scores could be affected by heightened anxiety, or difficulty with attention and language. Since all of these are hallmarks of the PTSD disorder, it is not surprising that the PTSD group had a slightly lower score. The impact of PTSD symptomatology has, in fact, been confirmed in the literature [44] and indicates that these lower test score should not be interpreted automatically as having a lower IQ, per se. However, some research has shown that higher premorbid IQ may help protect against the onset of PTSD in response to traumatic events [6].

### Structural imaging

Cortical thickness (CT) was found to be thinner on average in the PTSD group. Since CT reflects the number, density, and size of neurons [9, 42], a reduction in CT may be interpreted as loss of dendritic spines or a change in the cortical mantle due to a decrease in neuronal number [22, 47]. A closer look at specific regions, using the lobe-based analysis, revealed that this thinning was primarily affecting the frontal and temporal gyri. A previous study on CT of veterans with PTSD showed thinning in the same gyri [22], confirming our observations. The role of these gyri in verbal learning and memory associates them with some cognitive deficits seen in this disorder [11, 55, 60].

Our cortical thickness analysis also showed thinning in the angular and lingual gyri. These are gyri that are known to play an integral role in organizing language and thoughts [4], which are key to performance on the WASI test and which could, therefore, also explain the lower test scores. A recent study on IQ scores and their neural correlates supports this speculation, as they found an association between CT of various regions, including the lingual gyrus, and scores on verbal and performance tasks [37]. The CT reduction in the angular gyrus could be attributed to diminished structural integrity, as suggested by reports of reduction in fractional anisotropy as well as regional cerebral blood flow that were found in that gyrus in participants with PTSD [10, 46].

The result of cortical thinning of the precentral gyrus and supplementary motor area was interesting, as it has not been reported in the literature. Nonetheless, a functional-MRI study with PTSD participants revealed a significant decrease in activity in the dorsal attention system, which includes the precentral gyrus and supplementary motor area [14, 38]. Such decrease in function manifests as difficulties with attention, as seen in this disorder. Due to the tight link between neural function and structure, reduced use could potentially explain the reduction in thickness in these areas [27]. However, this speculation needs to be investigated further in future studies.

The lobe-based analysis was further supplemented by a vertex-based analysis, which allowed identification of more localized changes in cortical thickness. This analysis revealed thinning within a number of cortical regions. For example, the left superior frontal gyrus, which the lobe-based analysis found to be overall thinner in the PTSD, showed smaller regions of localized thinning scattered across the gyrus. The lobe-based result reflects the effect of averaging of all local changes across the gyrus and, in this case, indicates that the thinning outweighed the thickening in extent and/or magnitude. In the case of other regions, including the precuneus and parahippocampal gyri, while local variations in thickness were found with the vertex-based analysis, those were averaged out in the lobe-based method, revealing insignificant differences in those regions. Thus, this indicates that there are numerous small-scale morphological changes across the cortex, which are responsible for the overall cortical thinning seen in PTSD soldiers.

Our hippocampal analysis revealed no significant reduction in the relative volume of the right and left hippocampi. This finding is difficult to reconcile with some of the literature [23, 24, 35], which reports bilateral hippocampal reduction. However, other studies of the hippocampi in PTSD are inconsistent with this view failing to replicate such findings [3, 19, 26, 33, 39, 56]. This may be attributed to recruitment of participants with different PTSD triggers (combat, abuse, accidents), number of exposures post the triggering event, age of onset and duration, severity of symptoms, exposure to medication, psychiatric treatment and the use of non-matched control groups. Future studies should attempt to isolate these various parameters and determine in what cases hippocampal volume is altered in PTSD.

The subcortical analysis revealed relative volumetric reduction of the caudate, and although the caudate has not received much attention in the literature, other studies have reported a decrease in its volume in PTSD [21, 25]. In contrast to these findings functional studies have reported normal caudate function in PTSD [18, 43, 54]. In depression, a common co-morbid symptom in PTSD, an association was found between depression severity and lower caudate volumes [41]. Taken together, these findings suggest disruption to the reward processing circuitry, and are consistent with a theoretical model on the neurocircuitry of stress and anxiety disorders [25, 49], which posits that a hypoactive ventromedial prefrontal cortex lacks sufficient inhibitory control/modulation of the amygdala. The amygdala, in turn, exhibits functionally-atypical responses that tend to be hyperactive in PTSD, and these aberrant circuitry interactions may be responsible for the heightened fear responses prevalent in the disorder. Whilst the directionality of the network effects that are responsible for abnormal responses in PTSD is unknown (in other words, whether the amygdala is solely overactive, leading to *relatively* decreased frontal activity, or an underactive frontal region fails to suppress amygdala responses, or a combination of the two), this interplay between regions likely leads to the problems seen with heightened responsivity to threat and altered emotional control [36, 49].

The role of cerebellar function in PTSD has been investigated in the literature. Increased activation in the cerebellum was found in PTSD patients exposed to traumatic reminders [17, 20]. One study to examine the cerebellar morphology in PTSD was conducted on paediatric maltreatment-related PTSD subjects [16], and found that overall cerebellar volume was decreased. Another study, using voxel-based morphometry, reported greater gray matter density in the cerebellum of rape victims with PTSD compared to trauma-exposed healthy controls [51]. Our analysis would be consistent with the latter study, as we show a volumetric increase that was localized to lobules 7b, 8a–b. This difference may be attributed to the age at exposure to the traumatic event and age of onset of PTSD. In childhood the cerebellum is undergoing a rapid developmental change, which could potentially lead to an impact on its neurobiological and developmental processes. In general, our finding of larger cerebellar volumes in adult *combat*-*related* PTSD is novel. Lobules 7b and 8a–b play an important role in emotional processing [1], which is known to be affected in combat-related PTSD [52]. Future work should include measures of childhood trauma history in both groups, with and without PTSD.

In all of our analyses, we examined group-by-age interaction effects. Most notably we found such interaction in the case of the right and total cerebellar volumes, as well as in cerebellar lobules 3, 5, crus 1–2. The meaning of these results is not immediately clear, particularly as age did not correlate with age of onset of the disorder. A future study will attempt to elucidate this and shed light on the role of age of diagnosis as well as duration of the disorder as a function of neuroanatomical changes in PTSD. The paediatric study did see a correlation between duration of the trauma that led to the PTSD, age-of-onset of PTSD, and cerebellar volume [16]. Future studies should assess whether these also play a role in adult-onset PTSD.

Whilst not examined here, the role of stress-related neurochemical changes on brain structure and function has been investigated previously. It is theorised that increased stress-related glucocorticoid release may induce apoptosis and thus, a loss of neural tissue, which would lead to altered brain morphology, such as cortical thinning (see [5, 58, 61] for a review). Functional changes, that could be a consequence of subtle structural abnormalities due to biochemical dysregulation, are also known in the disorder; these include altered catecholamine levels (that include dopamine and norepinephrine), modulating HPA axis response, leading to increases in fear conditioning and arousal states [48].

## Limitations

These results should be interpreted with some caveats. First, it is possible that early life trauma could have at least in part contributed to the effects seen here. Whilst this was not quantitatively measured or explicitly controlled for in our analysis, the CAF psychiatrist conducting examinations of our cohort carefully screened potential participants, and ensured that those displaying potential PTSD symptomology could trace the nature of their triggering event was to a combat-related experience whilst on deployment. This psychiatric evaluation would at least attenuate any confounding factor, as we can be confident the primary reason for developing PTSD was due to combat-related operations. In future studies, evaluation of lifetime trauma will be measured to categorically evaluate the contribution of such events to any observed structural effects.

Second, it is not possible to say whether psychological stress due to experiential trauma is responsible for these morphological observations, and/or whether pre-existing structural deficits are a contributing risk-factor for the disorder. Animal studies have shown that chronic stress leads to alterations in the structural properties of the vmPFC (see review [40]), and studies of non-combat- and combat-exposed twins have shown that the pre-morbid hippocampal volume might to explain variability in susceptibility to the disorder [23], although the fact we observed no differences in this region makes it difficult to reconcile with the literature. Other studies have shown that alterations in ACC structure are likely caused by trauma-exposure [30], and similarly, it is possible that combat-exposure leads to modifications of the anatomical properties, such as those observed here. Thus, we cannot yet determine whether the anatomical effects reflect pre-morbid atypicalities that relate to the susceptibility of PTSD for those placed in stressful, military situations, or whether these effects are caused by such exposure.

Third, whilst the PTSD group did display evidence of secondary psychiatric symptoms, PTSD was defined as the primary diagnosis by the CAF psychiatrist. PTSD is a disorder based on clinical symptoms that include anxiety (with until recently the disorder being classified as an anxiety disorder in and of itself), and depression; therefore, if this PTSD group did not present higher scores on these disorder measures, it is not likely that they would have a PTSD diagnosis [59]. However, it is possible that the contribution of potentially confounding conditions may explain in part some of our findings.

## Conclusions

Soldiers with PTSD were found to have altered brain morphology that included reduction in cortical thickness particularly in frontal and temporal regions, decreased relative volumes of the caudate, but relative enlargement in several lobules within the cerebellum. Future studies will evaluate the role of age-of-onset and duration of disorder on these brain structures.

## Abbreviations

CLASP: Constrained Laplacian Anatomical Segmentation using Proximities
CT: cortical thickness
GM: grey matter
MAGeT: Multiple Automatically Generated Templates
MNI ICBM152: Montreal Neurological Institute International Consortium for Brain Mapping 152
MRI: magnetic resonance imaging
PTSD: posttraumatic stress disorder
WASI: Wechsler Abbreviated Scale of Intelligence
WM: white matter
